# Acute oxygen sensing by vascular smooth muscle cells

**DOI:** 10.3389/fphys.2023.1142354

**Published:** 2023-03-03

**Authors:** Alejandro Moreno-Domínguez, Olaia Colinas, Tarik Smani, Juan Ureña, José López-Barneo

**Affiliations:** ^1^ Instituto de Biomedicina de Sevilla (IBiS), Hospital Universitario Virgen del Rocío/CSIC/Universidad de Sevilla, Seville, Spain; ^2^ Departamento de Fisiología Médica y Biofísica, Facultad de Medicina, Universidad de Sevilla, Seville, Spain; ^3^ Centro de Investigación Biomédica en Red Sobre Enfermedades Neurodegenerativas (CIBERNED), Madrid, Spain

**Keywords:** acute O_2_ sensing, hypoxic pulmonary vasoconstriction, hypoxic arterial vasodilation, vascular smooth muscle, mitochondria, ion channels

## Abstract

An adequate supply of oxygen (O_2_) is essential for most life forms on earth, making the delivery of appropriate levels of O_2_ to tissues a fundamental physiological challenge. When O_2_ levels in the alveoli and/or blood are low, compensatory adaptive reflexes are produced that increase the uptake of O_2_ and its distribution to tissues within a few seconds. This paper analyzes the most important acute vasomotor responses to lack of O_2_ (hypoxia): hypoxic pulmonary vasoconstriction (HPV) and hypoxic vasodilation (HVD). HPV affects distal pulmonary (resistance) arteries, with its homeostatic role being to divert blood to well ventilated alveoli to thereby optimize the ventilation/perfusion ratio. HVD is produced in most systemic arteries, in particular in the skeletal muscle, coronary, and cerebral circulations, to increase blood supply to poorly oxygenated tissues. Although vasomotor responses to hypoxia are modulated by endothelial factors and autonomic innervation, it is well established that arterial smooth muscle cells contain an acute O_2_ sensing system capable of detecting changes in O_2_ tension and to signal membrane ion channels, which in turn regulate cytosolic Ca^2+^ levels and myocyte contraction. Here, we summarize current knowledge on the nature of O_2_ sensing and signaling systems underlying acute vasomotor responses to hypoxia. We also discuss similarities and differences existing in O_2_ sensors and effectors in the various arterial territories.

## 1 Introduction

Blood oxygen tension (PO_2_) is an important local factor in the regulation of circulation (Wadsworth, 1994; López-Barneo et al., 1999; Weir et al., 2005; Jackson, 2016). Arteries and arterioles are able to sense changes in PO_2_ in the blood or surrounding tissues and acutely respond with a vasomotor response (constriction or dilation) that depends on the vascular territory. Resistance pulmonary arteries constrict in response to a decrease in alveolar PO_2_ (alveolar hypoxia), thereby diverting blood flow from poorly ventilated lung regions towards areas with a higher PO_2_ to optimize ventilation/perfusion ratio. In conditions of persistent hypoxemia (low blood PO_2_), hypoxic pulmonary vasoconstriction (HPV) contributes to the pathogenesis of pulmonary hypertension (Harder et al., 1985; Weir et al., 2005; Waypa et al., 2006; Sylvester et al., 2012; Sommer et al., 2016). Unlike resistance pulmonary arteries, conduit pulmonary vessels and most systemic arteries (in particular in the skeletal muscle as well as in coronary and cerebral territories) dilate in response to hypoxia. Hypoxic vasodilation (HVD) also has a relevant physiologic role as it favors the perfusion of O_2_-deprived tissues (Detar, 1980; Marriott and Marshall, 1990; Wadsworth, 1994; Weir et al., 1997; Jackson, 2016).

Despite their considerable physiological relevance, the mechanisms underlying HPV and HVD have remained elusive and are still under debate. While substances released from the vascular endothelium (such as nitric oxide) can modulate vessel diameter, numerous studies have nevertheless demonstrated the existence of endothelium-independent hypoxic vasomotor responses and suggested the existence of an acute O_2_ sensor in vascular smooth muscle (VSM) (Hales, 1985; Madden et al., 1992; Pearce, 1995; Jackson, 2016). Indeed, a growing body of experimental work indicates that, in the case of HPV, the intrinsic O_2_ sensing mechanism in VSM involves the mitochondria, which appear to function as O_2_ sensors by initiating a redox-signaling pathway that leads to the activation of downstream effectors regulating vascular tone (Weir et al., 2005; Waypa et al., 2006). In this review, we briefly examine the most important mechanisms by which different VSM cells detect and respond to acute changes in O_2_ tension, focusing on the similarities and differences between the sensing and signaling pathways underlaying HPV and HVD. Given the limitations of scope and space, many relevant aspects of the extensive literature on the role of VSM in the pathophysiology of pulmonary and systemic vessels are considered sparingly, with readers referred to cited papers for a more in-depth explanation.

## 2 Hypoxic pulmonary vasoconstriction

### 2.1 General description

HPV is a fundamental homeostatic mechanism that evolved to generate acute adaptive compensatory responses to regional changes in ventilation of the lung. HPV is better observed in the most penetrating and smaller caliber resistance arteries close to the alveolar region, although other segments of the pulmonary circulation (including the pulmonary veins) also constrict in response to hypoxia. In contrast, hypoxia induces vasodilation in the proximal conducting vessels (main trunk and first branches of the pulmonary artery), as it does in the systemic vasculature. HPV is rapidly and reversibly triggered within a few seconds in response to hypoxia (Madden et al., 1992; Archer and Michelakis, 2002). The vasomotor response exhibits two phases when alveolar hypoxia is maintained: an initial acute phase involving rapid vascular contraction and an increase in vascular resistance, with subsequent partial relaxation lasting about 30 min. This phase is followed by a second phased involving a sustained increase in vascular tone. Both phases of HPV have been described in terms of changes in pulmonary arterial pressure at constant blood flow in isolated rat lungs or pulmonary lobes, as well as *via* the measurement of the isometric force exerted by isolated pulmonary arteries (Bennie et al., 1991; Leach et al., 1994; Weissmann et al., 1995; Weissmann et al., 2004; Robertson et al., 2001) (for an extended review see Sylvester et al., 2012). Although some studies have suggested that endothelium-derived vasoactive substances mediate HPV (Holden and McCall, 1984), the role attributed to the pulmonary vascular endothelium in HPV is largely regulatory (for an extended review see Grimmer and Kuebler, 2017). This notion is based on the observations that isolated myocytes (Madden et al., 1992; Zhang et al., 1997; Sham et al., 2000) and endothelium-denuded pulmonary arterial rings (Yuan et al., 1990; Bennie et al., 1991; Archer et al., 1998) are capable of contracting when exposed to hypoxia, indicating that HPV is a mechanism intrinsic to pulmonary artery smooth muscle cells (PASMCs). However, the magnitude of the pressor response can be modulated by different circulating mediators from the vascular endothelium and the autonomic nervous system (Archer et al., 1989; Frostell et al., 1991; Brimioulle et al., 1997; Weissmann et al., 2001).

### 2.2 O_2_-sensing mechanisms of pulmonary artery smooth muscle cells

The HPV reflex implies the presence of a functional acute O_2_-sensitive system in PASMCs. This sensor is able to detect decreases in alveolar O_2_ and to signal effector elements responsible for triggering membrane depolarization and extracellular Ca^2+^ entry (and eventually Ca^2+^ release from intracellular stores), resulting in a subsequent increase in vascular tone. In what follows, we briefly summarize the sensing/signaling systems reported so far which, although subject to debated, seem to have garnered experimental support.

#### 2.2.1 AMP kinase

The levels of alveolar hypoxia capable of triggering HPV do not seem to inhibit mitochondrial cellular respiration to a degree sufficient to compromise ATP production (Waypa and Schumacker, 2002; Sommer et al., 2016). Nonetheless, it has been suggested that small changes in the cytosolic AMP/ATP ratio could act as a signaling mechanism, inducing the activation of AMP kinase with a constant intracellular ATP concentration. This, in turn, would contribute to the release of Ca^2+^ from intracellular stores *via* cyclic ADP-ribose (cADPR, cyclic adenosine diphosphate ribose) through its interaction with ryanodine receptors (RyR) in the sarcoplasmic reticulum. Although it is not yet established whether AMP kinase directly elicits cADPR-dependent Ca^2+^ release, it has been proposed that this could result from phosphorylation by AMP kinase of ADP-ribosyl cyclase, which catalyzes the cyclization of NAD^+^ to produce cADPR, and a subsequent increase in cADPR accumulation. AMP kinase could also increase the sensitivity of the Ca^2+^ release process to cADPR due to the phosphorylation of RyRs or an intermediate cADPR-binding protein (Leach et al., 2000; Evans et al., 2005). As the production of cADPR is also correlated with NADH levels, accumulation of the two nucleotides in response to hypoxia would represent an AMP kinase-mediated O_2_-sensing and signaling system (Wilson et al., 2001). In contrast to this proposal, it has also been shown that selective inhibition of cADPR-induced Ca^2+^ release has no effect on the initial acute phase of HPV, suggesting that AMP kinase activation contributes mainly to the sustained phase of HPV (Dipp and Evans, 2001; Moudgil et al., 2005).

#### 2.2.2 NADPH oxidase

NADPH oxidase (NOX) is capable of producing reactive oxygen species (ROS) in proportion to PO_2_ levels by catalyzing the transfer of electrons from NADPH to O_2_, and has therefore been suggested to work as an O_2_ sensor (Mohazzab et al., 1995; Marshall et al., 1996). However, although the loss of functional NOX dramatically lowers ROS production in NOX2-deficient mice, HPV is still preserved in these animals (Archer et al., 1999). Protein kinase C (PKC) has also been suggested to regulate HPV *via* NOX activation (Weissmann et al., 1999). In this regard, several reports in the literature described a possible direct interaction between mitochondria and NOX, where PKC-dependent ROS release from mitochondria could activate NOX under hypoxic conditions, leading to further increases in ROS levels, as well as ROS-dependent increases in intracellular Ca^2+^, and in PASMC contraction (Rathore et al., 2008). However, the direct effects of mitochondrial ROS on PKC activity have not yet been investigated. In contrast, it was proposed that glucose-6-phosphate dehydrogenase (G6PD), an enzyme that participates in the pentose phosphate pathway catalyzing the production of NADPH, is responsible for NOX activation in hypoxia, as G6PD-deficient mice exhibit a reduced HPV response (Gupte et al., 2010). In all of these cases, NOXs are considered to play an important role in HPV either as O_2_ sensors *per se* or as part of the hypoxic signaling cascade.

#### 2.2.3 Rho-associated protein kinase

Rho kinase is a major mediator of Ca^2+^ sensitization, capable of inducing PASMC contraction by inhibiting myosin light-chain phosphatase in a Ca^2+^-independent manner (Somlyo and Somlyo, 2003). Hypoxia, acting through Rho kinase, is capable of accentuating HPV by increasing phosphorylation of the light chain and augmenting contraction (Wang et al., 2001). Evidence that Rho kinase is involved in sustained HPV comes from studies in isolated arteries and in perfused rat lungs, where Y-27632, a Rho kinase inhibitor, preferentially inhibited the sustained contraction phase of HPV, but had no impact on the acute hypoxic contraction (Robertson et al., 2000; Wang et al., 2001; Fagan et al., 2004). The hypoxic activation of Rho kinase was suggested to be dependent on ROS, as superoxide could trigger Rho kinase-mediated Ca^2+^ sensitization and vasoconstriction in rat pulmonary arteries (Knock et al., 2009). However, the role of this kinase in eliciting a specific HPV response is unclear, as Rho kinase activity is also present in systemic vascular beds (Numaguchi et al., 1999; Mukai et al., 2001), which dilate in response to hypoxia. Therefore, Rho kinase seems to contribute to a modulatory system that determines the magnitude of HPV but is not part of the acute O_2_ sensing pathway.

#### 2.2.4 Mitochondria

There is general consensus that mitochondria in PASMCs play a major role as a sensor and signaling system of alveolar hypoxia. Given their high O_2_ consumption and ability to generate ROS, as well as their content in numerous metabolic intermediaries and proximity to the plasma membrane and the sarcoplasmic reticulum, mitochondria are ideal candidates for having an essential O_2_ sensing and signaling function (Weir et al., 2005; Waypa et al., 2010; Sommer et al., 2016). Mitochondria can work as early detection systems for the conjugation of O_2_ supply and demand, optimizing the ventilation/perfusion ratio through of HPV response in the case of pulmonary resistance arteries.

Mitochondria involvement in pulmonary vascular sensitivity to O_2_ was initially postulated after it was verified that certain proximal inhibitors of the electron transport chain (ETC) triggered an acute, hypoxia-like, pressor response in PASMCs (Rounds and McMurtry, 1981). On the other hand, changes in the equilibrium of redox pairs able to interact with sulfhydryl groups of proteins were proposed to contribute to the O_2_-dependent physiological control of pulmonary vascular smooth muscle tone (Weir et al., 1985). Subsequently, a mitochondrial-based redox O_2_ sensor in PASMCs was suggested by several investigators (Archer et al., 1993; Waypa et al., 2001; Waypa et al., 2010; Weissmann et al., 2003; Wang et al., 2007; Song et al., 2017). O_2_-regulated K^+^ channels (Post et al., 1992; Yuan et al., 1993), potentially modulated by redox changes, were also described in PASMCs (Archer and Michelakis, 2002; Cogolludo et al., 2006; Mittal et al., 2012; Olschewski and Weir, 2015). Although it is generally agreed that mitochondria-dependent redox changes mediate the effects of hypoxia on membrane ion channels, a long-standing debate has ensued on whether hypoxia increases or decreases mitochondrial ROS production as well as on what might be the source of Ca^2+^ required for myocyte contraction (either extracellular influx or the release from sarcoplasmic reticulum) (Michelakis et al., 2002; Waypa et al., 2006; Wang and Zheng, 2010; Veit et al., 2015). Using PASMCs transfected with genetically encoded fluorescent probes, it was shown that hypoxia increases ROS in the cytosol and the mitochondrial intermembrane space (IMS) but decreases ROS within the matrix (Waypa et al., 2010). In our view, discrepancies regarding the type of ROS signal generated in PASMCs during hypoxia are probably a consequence of the different methodologies used to measure ROS production by the cells and the various organ/cellular preparations studied. The various methods of ROS detection used can also be particularly sensitive to different radical species (e.g., O_2_^−^, H_2_O_2_ or OH^−^), which could interact in different ways with ion channels (Sahoo et al., 2014). In addition, experimental observations across numerous studies were based on the extracellular application of redox reagents which may not properly mimic the changes in intracellular ROS signals generated during hypoxia (Reeve et al., 1995; Michelakis et al., 2004; Sommer et al., 2010; Waypa et al., 2010).

In the context of the current discussion, it is relevant to mention that an integrated mitochondrial-to-membrane signaling (MMS) model was proposed to explain acute O_2_ sensing by chemoreceptor glomus cells in the carotid body (CB), the prototypical acute O_2_ sensing organ in mammals. CB glomus cells are innervated by sensory fibers connected to brainstem centers that trigger a reflex hyperventilatory response and increased cardiac output in response to hypoxemia. In the MMS model, hypoxia produces a decrease in the activity of cytochrome c oxidase (CCO), thereby leading to a backlog of electrons along the ETC, with an increase in the reduced quinone (QH_2_)/quinone (Q) ratio and a slowdown of mitochondrial complex (MC) I. These changes result in the accumulation of NADH and production of a rapid and reversible increase in ROS at the mitochondrial intermembrane space (possibly H_2_O_2_ generated in MCI and MCIII). After equilibration with the cytosol, these signals modulate plasmalemmal ion channels to induce cell depolarization. Monitoring mitochondrial ROS production with the genetic probes described above for PASMC (Waypa et al., 2010) showed that, in parallel with the signaling increase in ROS at the IMS and cytosol, hypoxia produces a fast and highly reversible decrease in ROS at the mitochondrial matrix, which is interpreted as a non-selective signal derived from the activity of matrix dehydrogenases (Fernández-Agüera et al., 2015; Arias-Mayenco et al., 2018; Moreno Domínguez et al., 2020).

**FIGURE 1:**
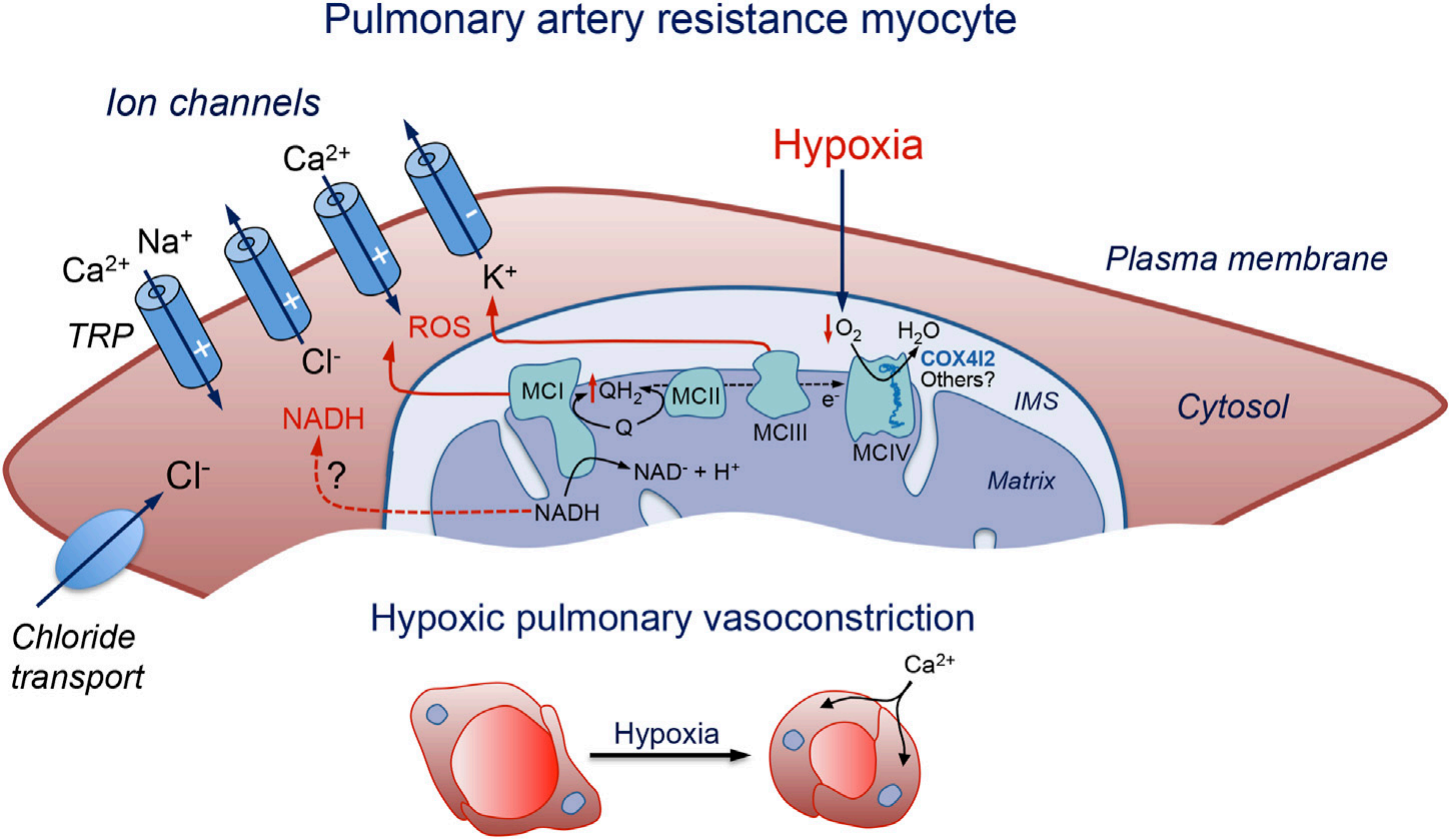
Schematic representation of a pulmonary artery resistance myocyte illustrating the molecular pathways proposed to be involved in acute pulmonary vasoconstriction. Hypoxia slows down the mitochondrial electron transport chain and causes the generation of signaling molecules (ROS and possibly NADH) which modulate membrane ion channels to produce cell depolarization, Ca^2+^ influx, and contraction. IMS: intermembrane space; MC: mitochondrial complex; NADH: reduced form of the nicotinamide adenine dinucleotide; Q: ubiquinone; QH_2_: reduced ubiquinone; ROS: reactive oxygen species; TRP: transient receptor potential channel. The symbols (−) and (+) indicate inhibition or activation of the channels during hypoxia, respectively. See text for further details.

The MMS model of acute O_2_ sensing was initially suggested given that mice deficient in NDUFS2, a core subunit essential for MCI assembly and NADH dehydrogenase activity (Kashani-Poor et al., 2001; Baradaran et al., 2013), showed selective abolition of CB glomus cell sensitivity to hypoxia and the HVR (Fernández-Agüera et al., 2015; Arias-Mayenco et al., 2018). Interestingly, it was also shown that silencing *Ndufs2* expression with interfering RNA (siRNA) selectively blocked the contractile response of PASMCs to hypoxia without affecting responses to other vasoconstrictor agents (Dunham-Snary et al., 2019). Moreover, mice lacking the gene coding the Rieske iron-sulfur protein (RISP) in cultured PASMC show a disruption of MCIII with inhibition of hypoxic ROS production and attenuation of the acute increase in right ventricular systolic pressure during hypoxia (Waypa et al., 2013). Recent studies on CB cells using the same mouse model showed a loss of both the hypoxic NADH and IMS ROS signals along with glomus cell responsiveness to hypoxia. However, in RISP-deficient mice as well as in MCI-deficient mice, modulation of the matrix ROS by hypoxia is maintained, suggesting that this signal is not directly involved in acute O_2_ sensing (Arias-Mayenco et al., 2018; Cabello-Rivera et al., 2022). It seems, therefore, that the mitochondrial-based O_2_ sensing process in PASMCs share many properties with the MMS model described for CB glomus cells (Figure 1). In agreement with this, mice without the gene coding for COX4I2, an atypical MCIV subunit isoform highly expressed in the lung and in glomus cells which may modulate the apparent affinity of CCO for O_2_, show a reduction of HPV (Sommer et al., 2017) as well as the hypoxic activation of CB glomus cells (Moreno-Domínguez et al., 2020). In addition to COX4I2, other MCIV subunits may contribute to modulating the sensitivity of mitochondria for O_2_ in PASMCs (Figure 1).

### 2.3 HPV effector elements

It is well established that HPV, at least the initial phase, requires PASMC membrane depolarization due to the modulation of several types of ion channels. Hypoxia-induced membrane depolarization leads to the opening of L-type Ca^2+^ channels, Ca^2+^ influx, and myocyte contraction. Numerous ion channel types expressed in the PASMC plasma membrane are susceptible to modification by the cellular redox state and have been identified as potential targets of hypoxic vasoconstriction downstream of the hypoxia-induced mitochondrial signals. Although ROS seem to be the main molecule signaling hypoxia in PASMCs, NADH accumulation (another mitochondrial signal generated during hypoxia in CB chemoreceptor cells (Moreno-Domínguez et al., 2020)), may also have a relevant signaling role. The most significant ion channel classes which are the target of the hypoxia-signaling pathway mediating HPV are described below.

#### 2.3.1 Potassium channels

Several groups have shown that acute hypoxia can inhibit voltage-gated (Kv) channel activity in dispersed PASMCs and reported a contribution of these channels to HPV (Post et al., 1992; Yuan et al., 1993; Hulme et al., 1999; Archer et al., 2004). Some studies have shown that members of the two-pore domain potassium channel family (e.g., TASK-1) are also inhibited by moderate hypoxia in human PASMCs (Olschewski et al., 2006), although these channels do not seem to be absolutely required for HPV of murine intra-pulmonary arteries (Murtaza et al., 2017). However, TASK-1 channels appear to play a prominent role in regulating membrane potential in human PASMCs since mutations in the *KCNK3* gene (coding TASK-1 channels) have been reported in patients with sporadic and familial pulmonary hypertension (see Lambert et al., 2018).

The O_2_-sensitive voltage-dependent K^+^ current in PASMCs has been proposed to be slowly inactivating, sensitive to 4-AP, and resistant to charybdotoxin. This profile would exclude several types of K^+^ channels (such as BK_Ca_, Kv1.2, Kv1.6, Kv1.4 or Kv4.3) as HPV mediators (Archer et al., 2004). The existence of several subtypes of PASMCs according to the density of Kv (delayed rectifier) and maxi K^+^ channel expression has been reported. Myocytes containing predominantly Kv channels are mostly found in resistance arteries, whereas conduit pulmonary arteries contain myocytes predominantly expressing maxi K^+^ channels or a mixture of Kv and maxi-K^+^ channels (Archer et al., 1996, 2004; Smani et al., 2001). Inhibition of Kv channels was shown to initiate membrane depolarization, activation of voltage-dependent Ca^2+^ channels, and vasoconstriction (Nelson and Quayle, 1995) (Figure 1). On the other hand, downregulation of Kv channels (e.g., Kv1.5) was associated with a depolarized PASMC phenotype and pulmonary hypertension (Yuan et al., 1998; Remillard et al., 2007; for review see Lambert et al., 2018). However, the mechanism of Kv inhibition under hypoxic conditions has not been fully elucidated. In line with the hypothesis suggesting that an increase in ROS concentration serves as an activating mechanism of HPV, some studies have verified that the application of H_2_O_2_ mimics the hypoxia-induced depolarization in mouse PASMCs, inhibiting the current mediated by Kv channels (Sommer et al., 2017). However, it must be kept in mind that H_2_O_2_ can interact with many different redox-susceptible sites in ion channels (Sahoo et al., 2014; Mori et al., 2016). As mentioned above, the extracellular application of H_2_O_2_ does not necessarily mimic the effect of this reagent when it is generated by intracellular ROS-producing organelles (see López-Barneo and Ortega-Sáenz, 2022). Intracellular NADH, which binds to Kv channel *ß* subunits with relatively high affinity (Kd 1 μM) (Liu et al., 2001), is known to inhibit the activity of several subclasses of Kv channels (Tipparaju et al., 2005; Kilfoil et al., 2019), that are modulated by hypoxia in pulmonary resistance myocytes (Archer et al., 1998; Moral-Sanz et al., 2016). However, whether NADH accumulation has a relevant role as a hypoxic signaling molecule in PASMCs remains to be determined.

#### 2.3.2 Transient receptor potential (TRP) channels

Ca^2+^-permeant cationic TRP channels, in particular TRPC1, TRPC6 and TRPV4, are highly expressed in resistance pulmonary myocytes where they contribute to Ca^2+^ entry and contraction (Remillard and Yuan, 2006; Reyes et al., 2018). TRPC channels can form complexes with Stim1/2 to mediate store-operated Ca^2+^ entry. The first, rapid phase of HPV, is abolished in TRPC6 knockout mice, while knockdown of Stim1 inhibits the second phase (Weissmann et al., 2006; Lu et al., 2009). Pharmacological blockade of TRPC6 channels also inhibits the acute phase of HPV (Urban et al., 2012). The relevance of TRPC6 channels for HPV is further supported by the fact that siRNA-mediated downregulation of TRPC6 expression inhibits the proliferation of PASMCs (Yu et al., 2003), while single nucleotide mutations of the *Trpc6* gene are associated with idiopathic pulmonary hypertension (Yu et al., 2009). A significant decrease in HPV is also observed in mice deficient of TRPV4 channels, which can form heteromeric assemblies with TRPC6 channels (Goldenberg et al., 2015). Direct activation of TRP channels secondary to the increase in ROS levels could constitute a complementary (or in some myocytes alternative) mechanism to K^+^ channel-mediated depolarization and cytosolic Ca^2+^ influx during hypoxia. TRP channel-mediated cationic currents may also produce myocyte depolarization and activate L-type Ca^2+^ channels (Sommer et al., 2016) (Figure 1). Acute O_2_ sensing by CB glomus cells is unaltered in mice lacking TRPC6 channels (Torres-Torrelo et al., 2021), which suggests that although pulmonary myocytes and CB chemoreceptor cells may share similar mitochondrial-based O_2_ sensing and signaling mechanisms, the effectors of the hypoxic response may differ in these cell types.

#### 2.3.3 Calcium channels

Voltage-dependent L-type Ca^2+^ channels play a leading role in HPV given that they mediate the rise in cytosolic [Ca^2+^] during hypoxia and in this manner couple PASMC excitation to contraction. Indeed, the L-type Ca^2+^ channel density in rabbit pulmonary resistance myocytes is almost twice that in conduit myocytes (Franco-Obregón and López-Barneo, 1996). However, it remains unclear whether Ca^2+^ channels are regulated by O_2_ tension or their activation during HPV is secondary to cell depolarization produced by hypoxia-induced modulation of other channel types (e.g., inhibition of K^+^ channels and/or activation of TRP channels) (Figure 1). The presence of two different types of myocytes in the pulmonary arterial tree whose voltage-gated Ca^2+^ channels are modulated by PO_2_ was previously reported. In this way, Ca^2+^ currents in conduit myocytes, which predominate in the main arterial trunk and large branches, are inhibited by low PO_2_, while in resistance myocytes, concentrated in the smaller arterial branches, hypoxia increases the amplitude of the Ca^2+^ current (Franco-Obregón and López-Barneo, 1996).

**FIGURE 2:**
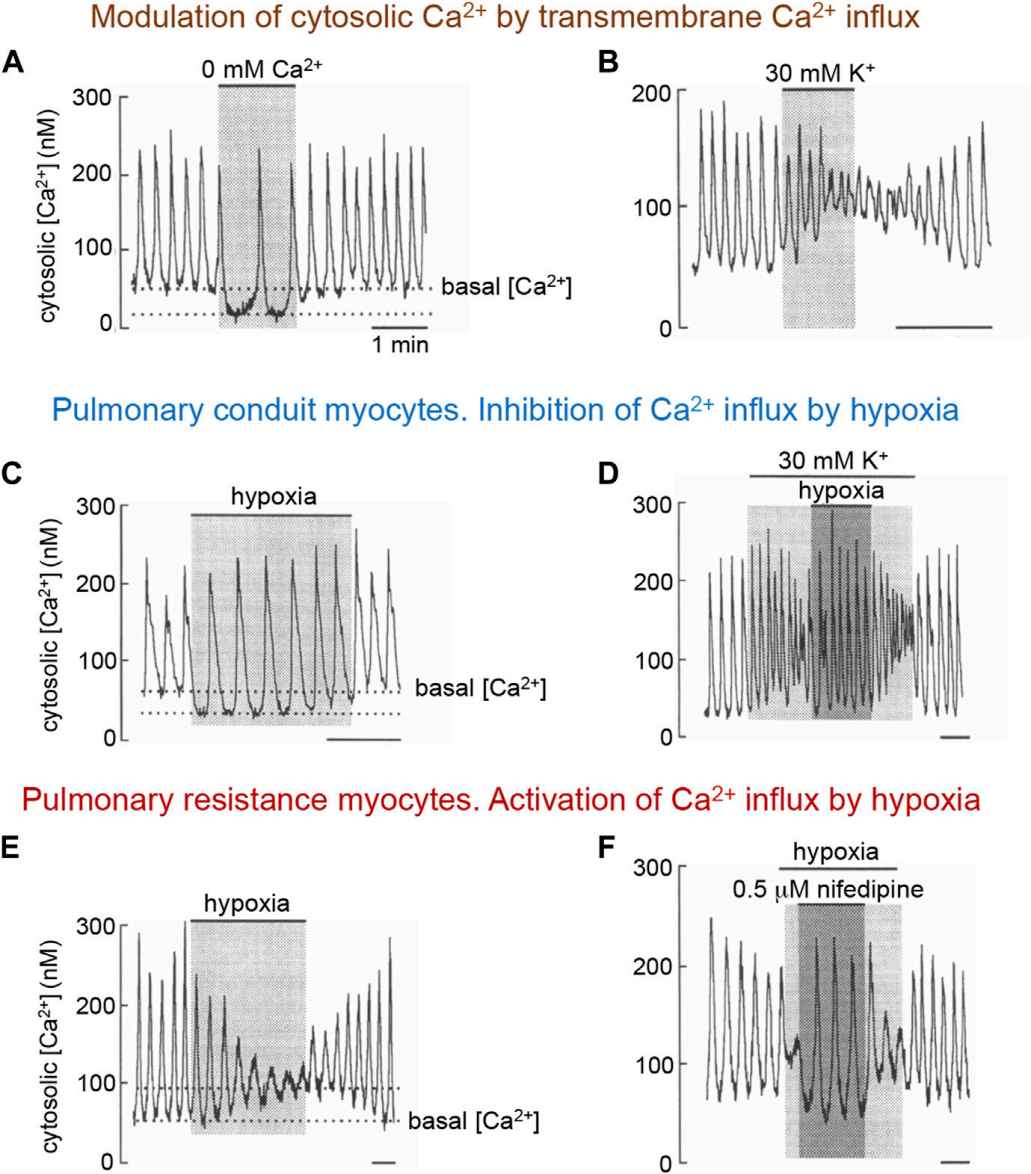
Modulation of spontaneous cytosolic Ca^2+^ spikes in rabbit pulmonary artery myocytes by transmembrane Ca^2+^ influx and hypoxia. **(A, B)**. Changes in basal Ca^2+^ and spike frequency and amplitude in a pulmonary resistance myocyte upon removal of extracellular Ca^2+^ or depolarization with high extracellular K^+^ (≥30 mM). Similar effects of Ca^2+^ removal or K^+^-induced depolarization are observed in pulmonary conduit myocytes. **(C, D)**, Decrease in basal cytosolic Ca^2+^ and changes in spikes characteristics in resting and depolarized conduit myocyte during exposure to hypoxia. **(E, F)**. Increase in basal cytosolic Ca^2+^ and changes in spike characteristics in a resistance pulmonary myocyte. Blockade of Ca^2+^ channels counteract the effect of hypoxia. In all figures application of hypoxia was done by switching from a bathing solution with PO_2_∼150 mmm Hg to another with PO_2_ ∼15 mm Hg. Time calibration bars in all panels is 1 min. Modified from Ureña et al., 1996.

The differential modulation by hypoxia of L-type Ca^2+^ channels in myocytes along the pulmonary arterial tree is strongly supported by the altered effects of low PO_2_ on cytosolic Ca^2+^ homeostasis in intact non-dialyzed VSM cells from the corresponding pulmonary arterial segments. As observed in other cells types, arterial myocytes (pulmonary and systemic) *in vitro* exhibit spontaneous oscillations of intracellular [Ca^2+^] (Ca^2+^ spikes), which largely represent the rapid release of Ca^2+^ from intracellular stores (Berridge, 1993). These Ca^2+^ spikes are modulated by transmembrane Ca^2+^ influx through L-type Ca^2+^ channels (see Blatter and Wier, 1992; Berridge, 1993; Franco-Obregón et al., 1995; Ureña et al., 1996). The removal of extracellular Ca^2+^ produces a rapid decrease in basal cytosolic [Ca^2+^] accompanied by either a decrease in the frequency of spikes or even their complete abolition (Figure 2A). In contrast, exposure of the cells to high external K^+^, presumably causing membrane depolarization and Ca^2+^ entry, produces an elevation in basal cytosolic [Ca^2+^], which is accompanied by a slight augmentation in Ca^2+^ spike frequency and a decrease in amplitude, or even suppression, of the spikes (Figure 2B). In most pulmonary conduit myocytes, as well as in myocytes of systemic arteries (see Section 3.3.2), hypoxia has a similar effect to that of removal of external Ca^2+^ (Figure 2C). The action of hypoxia on conduit myocytes is also opposite to that of high K^+^ (Figure 2D). In resistance myocytes, hypoxia raises basal cytosolic Ca^2+^ with a decrease in spike amplitude (Figure 2E); these effects are countered by the application of nifedipine (Figure 2F). The mechanism by which lowering PO_2_ differentially regulates L-type Ca^2+^ channels expressed in PASMCs is not known. It has been reported that the pore-forming subunit of L-type channels (α1C or Cav 1.2) expressed in the heart has specific cysteine residues whose oxidation could increase the probability of channel opening (Hool, 2015; Muralidharan et al., 2016). This fact would explain the increase in voltage-dependent Ca^2+^current in pulmonary resistance myocytes. It is possible that in conduction myocytes, or in myocytes of systemic arteries (see Section 3.3.2) Ca^2+^ channels are formed by *α*-subunit variants or auxiliary subunits that modify their sensitivity to mitochondrial signals (ROS and NADH).

#### 2.3.4 Chloride channels

**FIGURE 3:**
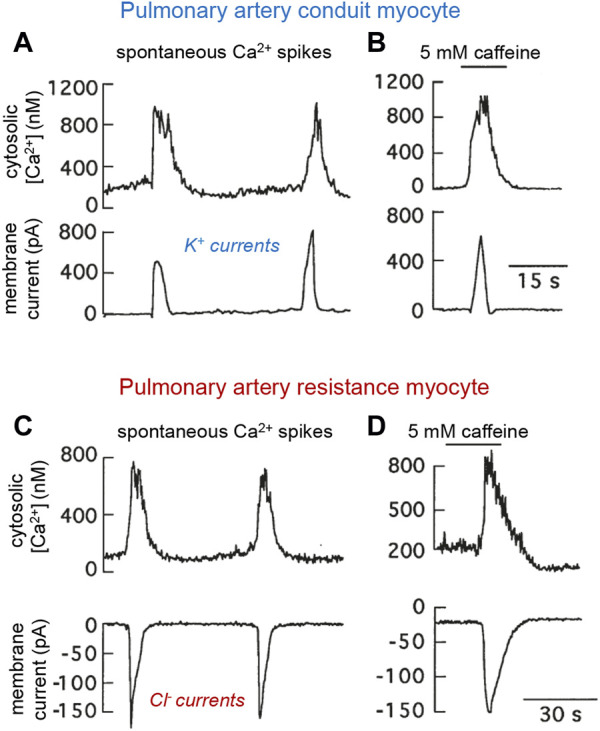
Spontaneous and caffeine-induced spikes of cytosolic Ca^2+^ and parallel modifications of transmembrane ionic currents in patch clamped rabbit pulmonary arterial myocytes. **(A, B)**. Conduit myocyte that generated outward K^+^ currents. **(C, D)**. Resistance myocyte in which the Ca^2+^ oscillations and caffeine induced inward Cl^−^ currents. Experiments done using the standard external solution and an internal solution containing Fura-2 and 22 mM ClNa. In all recordings membrane potential was −58.9 mV. Caffeine was added to the external solution. Modified from Smani et al., 2001.

The intracellular Cl^−^ concentration in VSM cells is unusually high (∼50 mM or even higher) due to active Cl^−^ transport, with the Cl^−^ equilibrium potential (near 0 mV) in these cells being more positive than the resting potential (∼−50 to −60 mV) (Yuan, 1997; Smani et al., 2001; Hübner et al., 2015; Leblanc et al., 2015). Therefore, activation of Cl^−^ channels normally leads to Cl^−^ efflux and cell depolarization, causing increased intracellular Ca^2+^ and contraction. Over the last few years, Cl^−^ channels have gained prominence as contributors to HPV and pulmonary hypertension. Ca^2+^-activated Cl^−^ channels are highly expressed in resistance PASMCs, where spontaneous cytosolic Ca^2+^ spikes or the pharmacologically-induced release of Ca^2+^ from internal stores generate inward (depolarizing) Cl^−^ currents. In contrast, increases in cytosolic [Ca^2+^] most frequently produce outward (hyperpolarizing) K^+^ currents in conduit myocytes (Smani et al., 2001) (Figure 3). These data suggest that hypoxic vasoconstriction in distal PASMCs due to Ca^2+^ influx or Ca^2+^ release from internal stores is potentiated by Cl^−^ channel activation. In favor of the prominent role played by Cl^−^ channels in HPV and the development of pulmonary hypertension are the upregulation by sustained hypoxia of Ca^2+^-activated Cl^−^ channels in PASMCs (Sun et al., 2012) along with the induction of Cl^−^ transporters and increased intracellular [Cl^−^] in chronically hypoxic rats (Sun et al., 2021). Moreover, the expression of TMEM16A, a member of the transmembrane protein 16 family which mediates most of the Ca^2+^-activated Cl^−^ current in PASMCs (Manoury et al., 2010), is upregulated in pulmonary hypertension and contributes to the enhanced pulmonary vasoconstriction associated with this pathological condition (Forrest et al., 2012; Leblanc et al., 2015; Maurer et al., 2020; Sun et al., 2021).

Taken together, the available experimental evidence suggests that the effector pathways mediating HPV are complex, as they do not depend on the modulation of a specific ion channel type. However, the level of expression of the main ion channel classes mediating HPV is higher in resistance myocytes than in conduit myocytes. It seems that, as occurs in CB chemoreceptor cells (see López-Barneo and Ortega-Sáenz, 2022), signaling molecules produced during hypoxia (ROS and possibly also NADH among others) interact promiscuously with different types of ion channels. Interestingly, the primary effects elicited by hypoxia on some ion channels in resistance myocytes (e.g., cell depolarization or Ca^2+^ release from stores) are potentiated by the activation of other ion channel classes to jointly produce a robust increase in cytosolic [Ca^2+^] and contraction.

## 3 Arterial hypoxic vasodilation

### 3.1 General description

There is consensus that O_2_ is a potent vasoactive agent and a major regulator of systemic vascular tone *in vivo* (Jackson, 2016). In contrast with resistance pulmonary arteries, a decrease in PO_2_ elicits relaxation and vasodilatation in systemic arteries, which increases O_2_ supply to tissues (Detar, 1980; Marriott and Marshall, 1990; Wadsworth, 1994). HVD is a generalized response, although it is particularly potent in brain, heart and skeletal muscle arterial territories. Together with other mechanisms (Gordon et al., 2007) increased blood flow during hypoxia probably contributes to neurovascular coupling - a dilation of small blood vessels whereby O_2_ availability is increased in the most active brain regions. Similarly, relaxation is the most common effect of low PO_2_ on the coronary arteries, which transport oxygenated blood to the myocardium. This response is of critical physiologic importance because it contributes to adjusting the amount of O_2_ supplied to the working heart to meet its metabolic needs, as any imbalance between O_2_ delivery and demand can lead to myocardial damage. For O_2_ to participate in the local regulation of blood flow in these tissues, small arteries and arterioles in the microcirculation must be able to sense acute changes in PO_2_ and trigger HVD. The cellular location of the O_2_ sensor and the mechanism underlying the physiological response to low PO_2_ nevertheless remains largely unknown (Jackson, 2016).

### 3.2 O_2_-sensing mechanisms in the systemic vasculature

**FIGURE 4:**
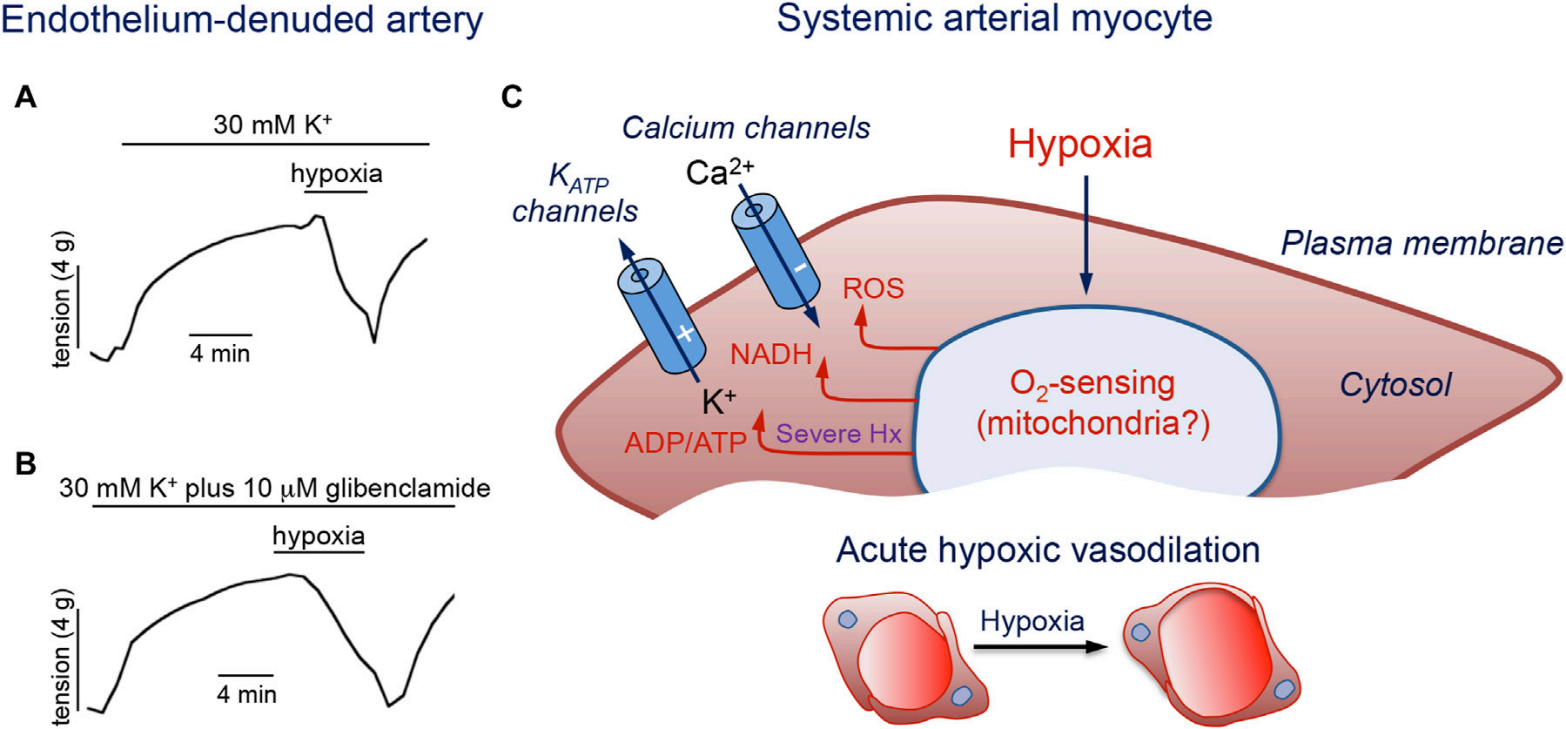
Molecular pathways involved in hypoxic vasodilation. **(A, B)**. Reduction of tension by hypoxia in endothelium-denuded porcine coronary arterial rings precontracted with 30 mM K^+^ or with 30 mM K^+^ plus 10 μM glibenclamide to block K_ATP_ channels. Modified from Smani et al., 2002. **(C)**. Schematic representation of a systemic artery myocyte. Hypoxia (Hx) acting on an O_2_ sensor generates intracellular signals (ROS, NADH and increased ADP/ATP ratio) that modulate membrane ion channels to produce cell hyperpolarization and inhibition of Ca^2+^ influx. The symbols (−) and (+) indicate inhibition or activation of the channels during hypoxia, respectively. See text for further details.

Different cellular sites, including the arteriolar muscular wall, the endothelial layer, erythrocytes, or extravascular cells, such as parenchymal and nerve cells, have been postulated to sense reductions in the PO_2_ levels and signal systemic arterial myocytes to elicit vasorelaxation (Jackson, 2016). It is well known that the vascular endothelium contributes to vasodilation by releasing potent relaxing factors that regulate smooth muscle contractility (Fredericks et al., 1994; Liu and Flavahan, 1997; Lynch et al., 2006). However, numerous studies have proposed VSM cells as a primary site for sensing decreased PO_2_ independently of metabolic factors released from the endothelium or surrounding tissues (Dart and Standen, 1995; Jackson, 2000; Smani et al., 2002; Gauthier, 2006). Indeed, it is well established that hypoxia can reversibly induce relaxation of endothelial-denuded arterial rings precontracted by exposure to high extracellular K^+^ (Smani et al., 2002) (Figures 4A,B).

**FIGURE 5:**
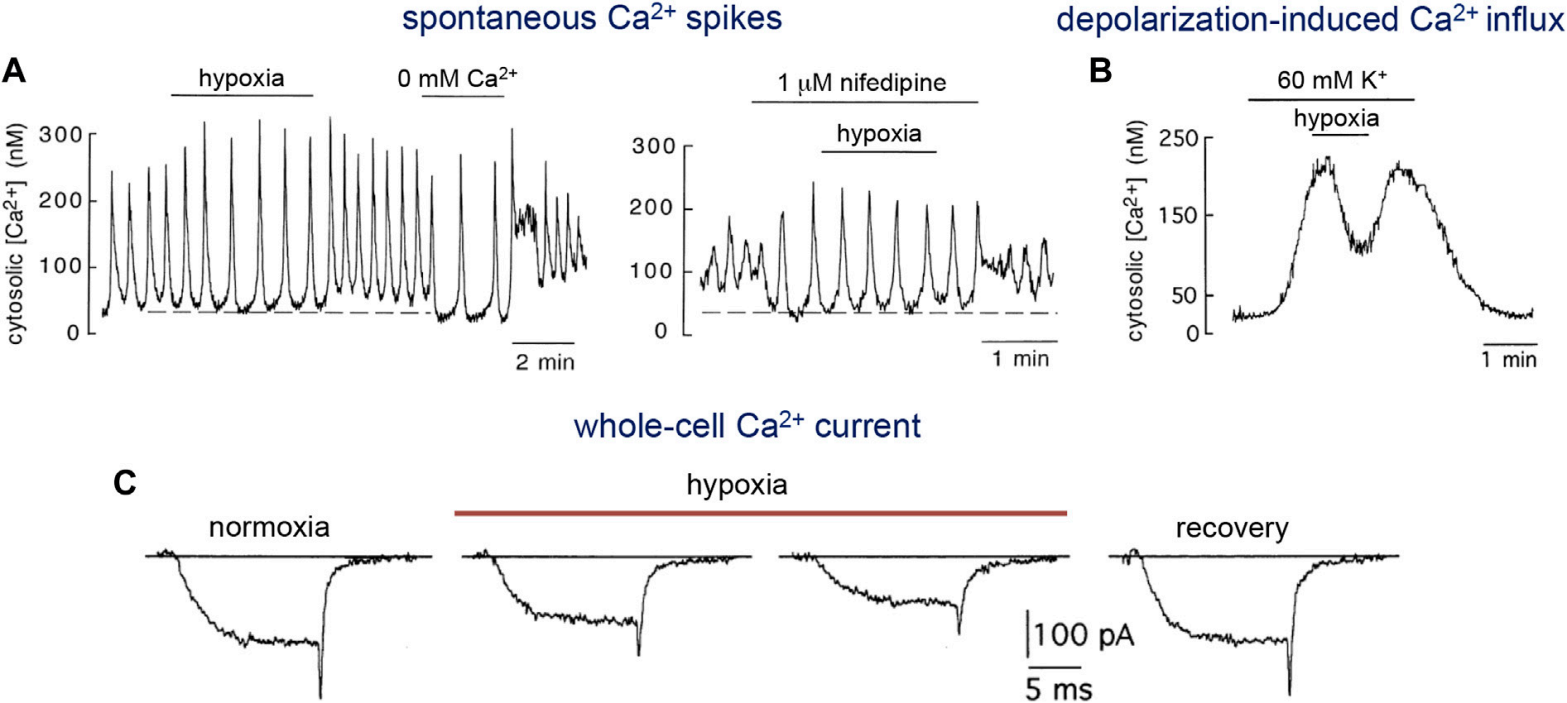
Inhibition of L-type Ca^2+^ channel activity by hypoxia. **(A)**. Response to hypoxia of a non-dialyzed human coronary artery myocyte which generated spontaneously intracellular Ca^2+^ oscillations. Left. Reduction of basal cytosolic Ca^2+^ (dotted line) and increase in the amplitude of the oscillations by hypoxia and removal of extracellular Ca^2+^ (0 mM Ca^2+^ and 1 mM EGTA added). Right. Blockade of L-type Ca^2+^ channels with nifedipine produced the same effect of hypoxia or the removal of external Ca^2+^. Note that hypoxia did not have any effect in the presence of nifedipine. Modified from Smani et al., 2002. **(B)**. Reversible reduction of cytosolic Ca^2+^ in a Fura-2 loaded quiescent pig coronary myocyte in which membrane voltage-dependent Ca^2+^ channels and Ca^2+^ influx were activated by depolarization with high extracellular K^+^. From López-Barneo et al., 1999. **(C)**. Ca^2+^ currents recorded from a rabbit celiac arterial myocyte during 15 ms step depolarizations to +10 mV from a holding potential of −80 mV. After exposure to hypoxia (PO_2_ ∼15 mmHg) there is a fast and progressive inhibition of current amplitude. Reversibility is illustrated by the recovery trace. Modified from Franco-Obregon et al., 1995.

As mentioned above (see Section 2.2.4), a growing body of work implicates the mitochondria as the major organelles responsible for acute O_2_ sensing and signaling in PASMCs, and similar mechanisms may also operate in systemic arterial VSM (Figure 4C). In this regard, it was reported that ROS generation increases during hypoxia in renal arteries (Michelakis et al., 2002). Experiments using genetically encoded roGPF sensors showed hypoxia-induced cytosolic and mitochondrial IMS ROS signals in systemic myocytes similar to those recorded in PASMC (Waypa et al., 2010). An oxidized stated with decreases in the [NADPH]/[NADP^+^] ratio was observed in hypoxic bovine coronary arteries, while HVD was attenuated by the thiol-reducing agent dithiothreitol (Gupte and Wolin, 2006). Therefore, although the mechanism of acute O_2_ sensing in systemic arterial myocytes is not known, it is likely that, as in the pulmonary circulation, mitochondria also play an important role. Since hypoxia has antagonistic functional effects on pulmonary and systemic VSM cells, the notion that both cell types share similar mitochondrial-based O_2_ sensing and signaling mechanisms would imply that they have differences in the downstream pathways and effector elements (Waypa et al., 2010). In this respect, it is relevant to note differences in ion channel types and densities between resistance and conduit pulmonary myocytes (see Section 2.3), given that acute hypoxic vasodilation is observed in conduit pulmonary arteries as well as in systemic arteries. Indeed, as shown in Figures 5A,B for single human and pig coronary arterial myocytes, hypoxia inhibits Ca^2+^ influx in a manner similar to that observed in conduit PASMCs (see Figures 2A–F). In coronary myocytes with spontaneous cytosolic Ca^2+^ oscillations, the effect of hypoxia is mimicked by the removal of extracellular Ca^2+^ (Figure 5A, left) or blockade of L-type Ca^2+^ channels with nifedipine (Figure 5A, right). Remarkably, hypoxia has no effect on cytosolic Ca^2+^ levels in the presence of nifedipine. In quiescent myocytes, hypoxia reversibly inhibits depolarization-induced Ca^2+^ influx (Figure 5B), thus further suggesting a direct action on the opening of Ca^2+^ channels (Figure 4C) (see below).

### 3.3 HVD effector elements

Over the last decades two major effectors have been associated with hypoxia-induced VSM relaxation: ATP-sensitive K^+^ (K_ATP_) channels and voltage-sensitive L-type Ca^2+^ channels. Membrane hyperpolarization caused by the activation of K_ATP_ channels or the inhibition of voltage-dependent Ca^2+^ channel opening can both lead to a decrease in Ca^2+^ influx and to VSM relaxation. However, these mechanisms may not be mutually exclusive, and interactions between them are highly probable (Pearce, 1995). In addition, other intracellular processes, including decreases in intracellular pH, elevations of inorganic phosphate, or activation of Kv7 channels (Taggart and Wray, 1998; Hedegaard et al., 2014), have also been suggested to contribute to hypoxic vasorelaxation.

#### 3.3.1 K_ATP_ channels

Numerous studies have suggested that K_ATP_ channels are involved in HVD in the skeletal and coronary circulations (Daut et al., 1990; Jackson, 1993; Marshall et al., 1993; Dart and Standen, 1995). However, other reports have shown that K_ATP_ currents remain stable during hypoxia because ATP concentrations do not drop to levels required for the channel activation (Jackson, 2000; Quayle et al., 2006). In agreement with these observations, it was also demonstrated that the hypoxia-induced decrease in resting tension of coronary or femoral arterial rings is unaffected by the inhibition of K_ATP_ channels with glibenclamide (specific inhibitor of K_ATP_ channels) (Smani et al., 2002; Quayle et al., 2006). In rat gracilis pressurized arteries, K_ATP_ channel blockers reduce membrane potential hyperpolarization and vasodilation in response to moderate and severe, but not mild, hypoxia (Frisbee et al., 2002). Currently, the most accepted view is that in most vascular territories intracellular ATP levels and the activity of K_ATP_ channels are not directly modified by physiological levels of hypoxia. However, K_ATP_ channel modulation by other paracrine and endocrine factors can contribute to the regulation of VSM cell excitability and contraction.

#### 3.3.2 Voltage-dependent Ca^2+^ channels

Several studies on systemic arterial smooth muscle have provided compelling evidence that HVD is triggered through mechanisms that decrease voltage-gated Ca^2+^ influx independently of K_ATP_ channel activation. As shown above (see Figures 4A,B), hypoxia induces acute relaxation of precontracted coronary arterial rings by mechanisms not involving the endothelium or K_ATP_ channel activation (Smani et al., 2002). In addition, inhibition of Ca^2+^ influx by hypoxia has been observed in intact, non-dialyzed myocytes from human, porcine and rabbit systemic arteries as well as in conduit PASMCs (see Figures 2A–F; Figures5A,B). The inhibition of L-type Ca^2+^ channels by hypoxia was initially demonstrated in patch-clamped freshly dispersed VSM cells from celiac arteries (Franco-Obregón et al., 1995). The effect of hypoxia on L-type Ca^2+^ currents is highly reversible and occurs within a physiological range of PO_2_ values without affecting currents mediated by T-type Ca^2+^ channels (Franco-Obregón et al., 1995; López-Barneo et al., 1999; Saitoh et al., 2006) (Figure 5C). To our knowledge, inhibition of L-type Ca^2+^ currents by hypoxia has been observed in VSM cells from several territories (celiac, femoral, coronary, cerebral, cheek pouch, and main pulmonary arteries) and species (human, pig, rabbit, rat and hamster), where hypoxemia normally produces vasodilatation (Marriot and Marshall, 1990; Pearce et al., 1992; Franco-Obregon et al., 1995; Herrera and Walker, 1998; Welsh et al., 1998; Smani et al., 2002; Quayle et al., 2006). Additionally, hypoxia decreases the amplitude of currents mediated by recombinant human L-type cardiovascular Ca^2+^ channel α1C subunit expressed in human embryonic kidney (HEK) cells in a manner indistinguishable from that observed in native smooth muscle L-type Ca^2+^ channels (Fearon et al., 1997). Moreover, inhibition of Ca^2+^ currents by hypoxia with characteristics similar to those reported in VSM cells has also been reported for rat and guinea pig ventricular myocytes (Hool, 2000; Rosa et al., 2012).

The mechanism whereby hypoxia modulates L-type Ca^2+^ channels is unknown although it seems that redox signals play a relevant role. Several groups have shown that whole-cell currents generated by recombinant Ca^2+^ channels (rabbit and human α1C subunit) expressed in mammalian cell lines are reduced in amplitude by lipophilic oxidizers of sulfhydryl groups, with these effects reverted by agents that reduce disulfide bonds (Chiamvimonvat et al., 1995; Fearon et al., 1999). Single channel recordings indicated that redox reagents alter channel gating without affecting permeation (Chiamvimonvat et al., 1995). These studies also reported that pre-treatment of the cells with oxidizing agents prevents inhibition of the Ca^2+^ current by hypoxia (Fearon et al., 1999). In agreement with these data, macroscopic Ca^2+^ currents and Ca^2+^ channel gating currents in guinea pig ventricular myocytes are inhibited by oxidants in a manner that is prevented by dithiothreitol (Lacampagne et al., 1995). These observations suggest that the pore-forming *α*-subunit of the cardiovascular L-type Ca^2+^ channel contains functionally important sulfhydryl groups that modulate gating. Studies performed using mutated α1C subunits with replacement of specific cysteine residues have shown that the L-type Ca^2+^ channels are indeed redox sensors although the effects of ROS on these channels are a matter of controversy (Hool, 2015; Muralidharan et al., 2016). Direct modification by oxidants of thiol groups in purified L-type Ca^2+^ channels reconstituted in proteoliposomes increases channel open probability, while reducing agents have the opposite effect (Hool, 2015). Therefore, it seems that redox reagents have different effects on Ca^2+^ channels reconstituted in bilayers compared with the same recombinant channel subunit expressed in mammalian cells (Chiamvimonvat et al., 1995; Fearon et al., 1999) or native Ca^2+^ channels (Lacampagne et al., 1995). It is possible that redox agents used in the different experimental conditions interact with distinct extra and intracellular cysteine residues existing in the α1C subunit which results in opposite effects on channel gating. It is also conceivable that ROS actions in cellular models are also mediated by intrinsic molecules associated with the α1C subunit.

Regarding the effect of hypoxia, it has been shown that L-type Ca^2+^ channels reconstituted in liposomes are insensitive to hypoxia, which suggests that, as discussed above, an independent O_2_ sensor (possibly mitochondria) is required for the hypoxic modulation of channel function. It is likely that besides ROS, hypoxia generates other signals (e.g., NADH) that also contribute to the physiological regulation of L-type Ca^2+^ channel gating. In this respect, it is relevant to note that hemeoxygenase (HO), a broadly distributed enzyme that degrades heme and produces CO, was been suggested to directly modulate cardiac L-type Ca^2+^ channels by binding directly to the C-terminal end of the α1C subunit (Rosa et al., 2012). Pharmacological CO production has also been shown to inhibit recombinant (α1C-subunit) Ca^2+^ channels expressed in mammalian cells, although the effect was proposed to be mediated by the ability of CO to promote production of ROS in mitochondria (Scragg et al., 2008). To our knowledge, vasomotor responses to hypoxia in HO knockout mice have not been studied; however, we have shown that HO-2 is not required for CB responsiveness to hypoxia (Ortega-Sáenz et al., 2006).

## 4 Conclusions and perspectives

Acute O_2_ sensing by VSM cells plays a fundamental role in the control of blood vessel diameter in response to the level of O_2_ tension in the nearby environment, this being a chemosensory function necessary to trigger compensatory adaptive responses. In this review we have focused on the study of two classic vasomotor responses to hypoxia in which our group has carried out considerable experimental work: HPV and HVD. HPV depends on the vasoconstriction of small caliber pulmonary arteries (resistance vessels) which contain a specific and varied set of plasmalemmal ion channels that trigger extracellular Ca^2+^ influx and contraction during hypoxia. Hypoxia can also directly or indirectly induce Ca^2+^ release from intracellular stores. Whilst much progress has been made during the last decades in defining the molecular processes underlying HPV, uncertainty remains about the O_2_-sensing mechanism and the signals that modulate ion channel activity. While it appears that mitochondria act as O_2_ sensors in pulmonary resistance myocytes, it remains to be unequivocally demonstrated, however, that ROS and other mitochondrial signals are directly responsible for modulating membrane ion channels. Moreover, although important advances have been made recently, the molecular determinants of mitochondrial specialization in lung arterial myocytes, in particular those responsible for the apparent low O_2_ affinity of cytochrome c oxidase, remain to be fully clarified. In addition to its physiological value, HPV is of pathophysiological interest since, if maintained, it can lead to pulmonary hypertension. Given the medical importance of this pathology, it is important to continue to make progress in understanding how the initial (acute) activation of myocytes by hypoxia leads to the cellular proliferation and vascular remodeling phenomena characteristic of pulmonary hypertension.

In contrast to resistance myocytes, myocytes in conduction pulmonary arteries relax with hypoxia; a response that is similar to the HVD seen in most systemic arteries. Although HVD has a fundamental physiological role, especially in the coronary and cerebral circulations, its molecular determinants remain poorly understood. The nature of the acute O_2_ sensing apparatus in systemic myocytes and how the sensor signals to membrane ion channels are still to be defined. Existing data suggest that inhibition of L-type Ca^2+^ channels by hypoxia plays a key role in HVD, but further experimental data are needed to definitively clarify this signaling pathway. The molecular description of HVD may provide new pharmacological targets of interest in clinical conditions such as coronary and cerebral ischemia or arterial hypertension. This pharmacological development may acquire even greater medical relevance if it is taken into account that inhibition by hypoxia of Ca^2+^ channels in cardiac myocytes, which share the pore-forming α1C subunit with arterial myocytes, may have a cardioprotective role in the event of cardiac ischemia. The fields of HPV and HVD face relevant and highly attractive challenges that are important in fundamental research, but which also have clear translational implications in cardiocirculatory pathophysiology and pharmacology, which must be addressed in future experimental work.
